# Rapid Capture of Cancer Extracellular Vesicles by Lipid Patch Microarrays

**DOI:** 10.1002/adma.202008493

**Published:** 2021-07-26

**Authors:** Hui‐Yu Liu, Ravi Kumar, Chunting Zhong, Saleh Gorji, Liliia Paniushkina, Ramsha Masood, Uwe A. Wittel, Harald Fuchs, Irina Nazarenko, Michael Hirtz

**Affiliations:** ^1^ Institute of Nanotechnology (INT) & Karlsruhe Nano Micro Facility (KNMF) Karlsruhe Institute of Technology (KIT) Hermann‐von‐Helmholtz‐Platz 1 76344 Eggenstein‐Leopoldshafen Germany; ^2^ Joint Research Laboratory Nanomaterials (KIT and TUD) at Technische Universität Darmstadt (TUD) Jovanka‐Bontschits‐Str. 2 64287 Darmstadt Germany; ^3^ Institute for Infection Prevention and Hospital Epidemiology Medical Centre Faculty of Medicine University of Freiburg Breisacher Straße 115 B 79106 Freiburg Germany; ^4^ Department of General and Visceral Surgery Centre of Surgery Medical Centre Faculty of Medicine University of Freiburg Breisacher Str. 86 79110 Freiburg Germany; ^5^ Physikalisches Institut & Center for Nanotechnology (CeNTech) Westfälische Wilhelms‐Universität Wilhelm‐Klemm‐Straße 10 48149 Münster Germany; ^6^ German Cancer Consortium (DKTK) Partner Site Freiburg and German Cancer Research Center (DKFZ) Im Neuenheimer Feld 280 69120 Heidelberg Germany

**Keywords:** breast cancer, dip‐pen nanolithography, extracellular vesicles, scanning probe lithography, supported lipid bilayers

## Abstract

Extracellular vesicles (EVs) contain various bioactive molecules such as DNA, RNA, and proteins, and play a key role in the regulation of cancer progression. Furthermore, cancer‐associated EVs carry specific biomarkers and can be used in liquid biopsy for cancer detection. However, it is still technically challenging and time consuming to detect or isolate cancer‐associated EVs from complex biofluids (e.g., blood). Here, a novel EV‐capture strategy based on dip‐pen nanolithography generated microarrays of supported lipid membranes is presented. These arrays carry specific antibodies recognizing EV‐ and cancer‐specific surface biomarkers, enabling highly selective and efficient capture. Importantly, it is shown that the nucleic acid cargo of captured EVs is retained on the lipid array, providing the potential for downstream analysis. Finally, the feasibility of EV capture from patient sera is demonstrated. The demonstrated platform offers rapid capture, high specificity, and sensitivity, with only a small need in analyte volume and without additional purification steps. The platform is applied in context of cancer‐associated EVs, but it can easily be adapted to other diagnostic EV targets by use of corresponding antibodies.

## Graphical abstract

Extracellular vesicles have great prospects as diagnostic and therapeutic tools. However, their isolation still poses challenges. A novel supported lipid‐membrane‐based platform is introduced for rapid capture, with high specificity and sensitivity, only a small needed analyte volume, and without additional purification steps. The platform is flexible and can be applied to many targets by corresponding antibodies.

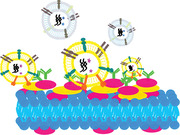

## 1 Introduction

Extracellular vesicles (EVs) are lipid‐bilayer enclosed structures shed by nearly all cells of a human body, containing abundant information about the cell of origin in form of proteins, RNA and DNA. They play an important role for cell–cell communication and present in all body fluids, like blood, urine, milk, tears, and others.^[^ ^1^, ^2^ ^]^ EVs are highly heterogeneous, and can be divided based on their origin into exosomes, microvesicles, and apoptotic bodies.^[^ ^2^, ^3^ ^]^ Exosomes are small EVs of 40–100 nm diameter released by the back‐fusion of multivesicular bodies (MVB) with the plasma membrane.^[^ ^4^, ^5^ ^]^ Microvesicles are a heterogeneous population of EVs with a diameter of ≈100–500 nm, budding directly from the cell membrane in response to various stimuli.^[^ ^3^ ^]^ Apoptotic bodies are products of the programmed cell death and are represented by a heterogeneous population of large vesicles with 400–3000 nm diameter, containing different components including residual organelles. Despite their different origin, exosomes, and microvesicles may harbor similar surface proteins and available isolation technologies do not allow their separation. Therefore, for both, the generic term “EV” was suggested in the community.^[^ ^2^ ^]^ In this work, we will focus on a population of small EVs and describe a new method for their capture. Consistent with the minimal information for studies of EVs (MISEV2018) guideline, we determine and characterize these vesicles as “extracellular vesicles (EVs),”^[^ ^2^ ^]^ and will use this term in this manuscript.

Recent achievements in cancer research demonstrated, that EVs play a highly important role in diseases progression, e.g., by organ‐specific preparation of the premetastatic niche.^[^ ^6^ ^]^ As cancer is the second most common cause of death in developed countries, cancer diagnosis and treatment became one of the most important tasks of modern medicine.^[^ ^7^ ^]^ However, despite of the progress and acquired knowledge, the imminent need for new tools for early detection of cancer and new therapeutic strategies remains challenging.^[^ ^8^, ^9^, ^10^ ^]^ Recent findings show that cancer cells produce significantly more EVs than healthy cells and use EVs for the regulation of the local tumor microenvironment as well as distant sites, promote vasculogenesis, immune suppression, tumor growth, and metastasis.^[^ ^11^, ^12^, ^13^, ^14^ ^]^ These cancer‐associated EVs are promising biomarkers for early cancer detection and therapy monitoring,^[^ ^15^, ^16^, ^17^, ^18^, ^19^ ^]^ as well as therapy itself,^[^ ^20^, ^21^ ^]^ and can be used in proteomics.^[^ ^22^ ^]^ However, as for their small size, high heterogeneity, and the complex background of other components present in body fluids, detection and isolation of EVs still pose a considerable challenge.^[^ ^19^, ^23^ ^]^ No clinically approved method for EV detection exists. In clinical use, such method should be accurate, specific, reproducible, rapid, easy to handle, cheap, and applicable for different types of EVs.^[^ ^24^ ^]^

## 2 Results and Discussion

### 2.1 The Lipid Microarray Based EV Capture Platform

**Figure 1:**
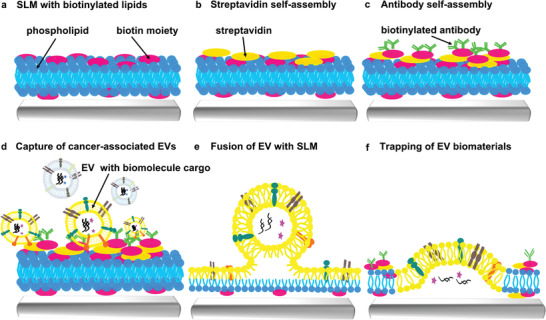
Scheme of extracellular vesicle (EV) capture by supporting lipid membranes (SLM). a) Fabrication of biotinylated SLM arrays by L‐DPN. b) Coating of the SLM arrays with streptavidin. c) Binding of biotinylated antibodies on the SLM arrays. d) Capture of cancer‐associated EVs on SLM arrays. e) Fusion of captured EVs with the SLM. f) Trapping of EV‐derived biomaterials (e.g., RNA, proteins) by the SLM.

In order to address the challenges of EV capture, we propose to exploit the unique interaction of biomolecules and biological membranes with biomimetic supported lipid membranes (SLMs)^[^ ^25^ ^]^ for a novel lipid microarray based strategy (**Figure** **1**). Two defining factors make SLMs ideal for this approach: First, they retain lipid dynamics (meaning the diffusion of lipid components within the membrane) which are the basis of many interactions in natural lipid membranes, such as signaling, transport and cell–cell contact. Second, pure lipid SLMs have intrinsic nonfouling properties (i.e., are protein and/or cell repellant).^[^ ^26^ ^]^ Both features allow the proposed SLM microarrays to become highly sensitive and accurate biosensors. In order to produce SLM microarrays, we employ lipid dip‐pen nanolithography (L‐DPN), which enables patterning of micro‐ and nanoscale‐sized lipid patches on various substrates in a multiplexed manner (i.e., different compositions of lipid within one array).^[^ ^27^, ^28^, ^29^, ^30^ ^]^ First, lipid membranes of 1,2‐dioleoyl‐sn‐glycero‐3‐phosphocholine (DOPC) with a 5 mol% admixing of the biotinylated phospholipid 1,2‐dioleoyl‐sn‐glycero‐3‐phosphoethanolamine‐*N*‐(cap biotinyl) (Biotin‐PE) are written via L‐DPN (Figure 1a). Then these are incubated with streptavidin solution to provide binding sites for biotinylated antibodies (ABs) (Figure 1b). Specific biotinylated ABs recognizing cancer‐associated EVs are incubated with the array and self‐assemble onto the lipid patches (Figure 1c). The ABs can also be delivered to individual patches by spotting techniques, in case multiplexed detection arrays are desired.^[^ ^31^ ^]^ When the arrays are exposed to liquids containing EVs carrying the targeted surface markers, these will attach to the lipid patches carrying matching ABs (Figure 1d). In addition to the mere attachment, the SLM‐based approach allows for membrane fusion taking place between captured EVs and the lipid patches in the array (Figure 1e). In natural membrane fusion,^[^ ^32^ ^]^ van der Waals attraction and/or membrane proteins bring two lipid membranes close together leading to increased membrane disorder, defects occurring in the bilayers then lead to final fusion.^[^ ^33^ ^]^ Albeit the molecular mechanisms of EV uptake by the target cells are not fully discovered, one of the possible mechanisms, suggested is fusion. To allow the EV to fuse with the cell membrane, an EV should be kept in a close proximity to the membrane of the recipient cell. It is suggested, that SNARE and Syncytin‐2 may be involved in the membrane fusion;^[^ ^34^, ^35^, ^36^, ^37^ ^]^ nonetheless, it is assumed, that contact of EV with the cell membrane may be sufficient to initiate fusion.^[^ ^38^, ^39^ ^]^ In our platform, the membrane bound ABs, therefore, are not only ensuring specificity of EV capture, but also promote membrane fusion and enhance the trapping processes, by holding the EV in near vicinity to the lipid patch. Finally, the membrane fusion process leads to trapping of EV cargo into the membrane patch (Figure 1f), which may open the route to further downstream analysis of the cancer‐related genome and proteome. The platform is free in choice of ABs and thus very flexible in the desired target. Recently, more and more biomarker‐proteins of cancer‐EVs are discovered,^[^ ^40^ ^]^ improving the precision and specificity of immunoaffinity‐based techniques for EV capture. Generally, these techniques are fast, efficient, and require only a small amount of analyte, making them applicable in clinical routine.^[^ ^41^ ^]^ ABs can be chosen from a library of available options to address specific needs in regard to the targeted EVs. Several tetraspanins, in particular CD9, CD63, and CD81, were found to be enriched on the majority of EVs and thus are frequently used as generic EV biomarker. In many cancer‐associated EVs, the epithelial cell adhesion molecule (EpCAM) was identified.^[^ ^15^ ^]^ Therefore, CD63 and EpCAM were selected in this study as general and as cancer‐specific EV biomarker respectively.

### 2.2 Capture of Cancer‐Associated EVs by SLM Arrays

**Figure 2:**
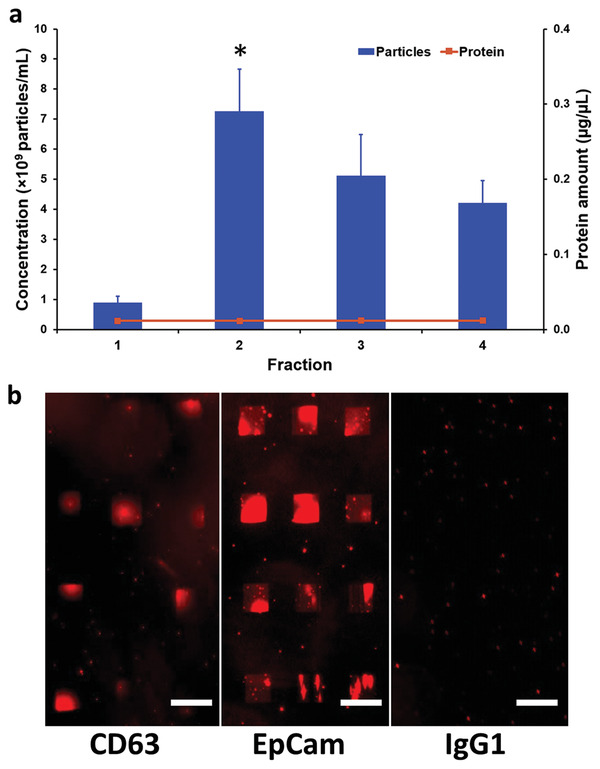
Capture of purified MCF7 EVs. a) Total protein amount (orange line) and concentrations of particles (blue bars) of fraction 1–4. Fraction 2 (marked with *) was chosen for subsequent experiments as it contains the highest number of particles and low protein concentration, confirming EV enrichment in these fractions according to the manufacturer recommendations. The error bars are given as the standard error of the mean in 4 independent samples. b) Consistent with the expectations, fraction 2 also leads to the strongest fluorescent signals (compared to the other fractions) on lipid patches harboring CD63 and EpCAM antibodies. Patches functionalized with IgG1 as isotype control show no fluorescent signal after incubation with fraction 2 (negative control). Scale bars equal 50 µm.

To establish a proof‐of‐concept for the platform, EVs from the conditional medium of the broadly used breast cancer line MCF7^[^ ^42^ ^]^ were utilized. To obtain standardized material for experimentation, small EVs were purified using size exclusion chromatography (SEC) and characterized according to the MISEV guidelines.^[^ ^2^ ^]^ The fractions 1–4 obtained from SEC were collected to determine the EV‐enriched fraction. For that, the protein content and number of particles were measured by micro bicinchoninic acid assay (BCA) and nanoparticle tracking analysis (NTA), respectively (**Figure** **2**a).

Following recommendations of manufacturer we selected fraction 2, exhibiting the highest number of particles and (as all fractions 1–4) a low amount of protein. In order to visualize the EVs in fluorescence microscopy, they were stained with a lipophilic dye (PKH26, red fluorescent), and incubated to lipid microarrays functionalized with CD63 and EpCAM ABs (Figure 2b). The lipid patches of the array exhibit red fluorescence in the presence of EVs bound either to CD63 or EpCAM, while lipid patches carrying isotype control ABs (immunoglobulin G (IgG)1) remain dark (Figure 2b). This shows that the lipid array can capture EVs by specific surface biomarkers (CD63 or EpCAM), while no fusion occurs when no matching biomarker is present on the EV (as seen on the sample containing the IgG1 isotype control). As the used dye is lipophilic, an additional control experiment was performed to ensure that the positive fluorescence signals on the lipid array are not caused by PKH dye not bound to EVs or detaching from the EVs and diffusing toward the array. For this, purified and stained EVs were incubated on a lipid array consisting of patches with pure DOPC on one side as negative control and Biotin‐PE/DOPC and AB functionalized patches on the other side. Here, as expected, only the AB‐functionalized patches emit the fluorescence signal, while the DOPC‐only patches remain dark (Figure S1, Supporting Information). This observation supports the specificity of the visualized fluorescence signal, indicating a binding of labeled EVs to the AB‐functionalized patches. Furthermore, this experiment highlights the antifouling property of the lipid layer, as the DOPC patches appear even darker then the surrounding nonfunctionalized glass‐slide surface, on which unspecific random adhesion of some of the stained EVs may take place. As an additional control, other SEC fractions were incubated on AB‐functionalized lipid microarrays. As expected, no significant fluorescence signal is observed on any of the patches functionalized with CD63, EpCAM ABs, or the IgG1 isotype control, supporting on the one hand the quality of EV isolation and on the other hand the specificity of the SLM‐based EV detection (Figure S2, Supporting Information). In combination, these results show that the lipid microarray platform can effectively capture EVs purified from the conditional medium of MCF7 cells as a proof‐of‐concept.

### 2.3 Specificity of EV Capture

**Figure 3:**
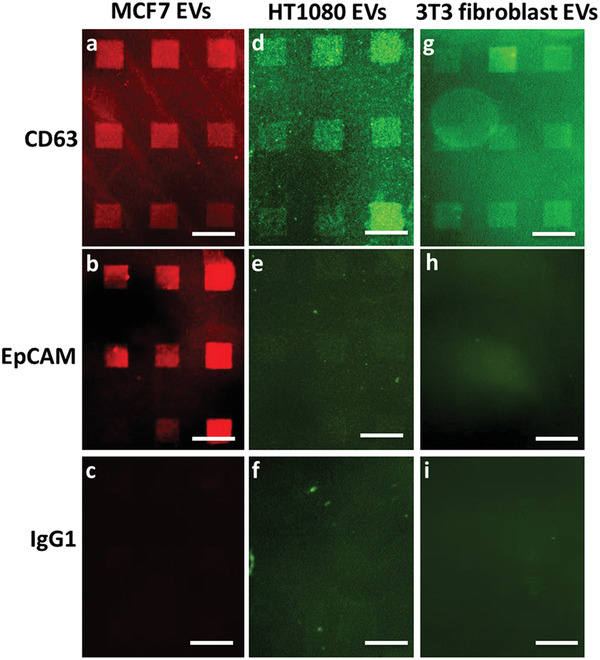
Specificity of EV capture. a,b) Fluorescently labeled (red, PKH26 dye) MCF7‐derived EVs are captured by CD63 (a) and EpCAM (b) antibody‐functionalized lipid patches. c) No capture is observed on lipid patches functionalized with IgG1 isotype control antibodies (negative control). d) Similarly, fluorescently labeled EVs (green, PKH67 dye) derived from HT1080 cells are captured by CD63‐antibody‐functionalized lipid patches. e) A minor signal of HT1080 EVs on the EpCAM‐functionalized lipid patches was detected, indicating that high EV concentration may cause some unspecific capture. f) Again, no interaction with IgG1 isotype control antibodies carrying lipid patches is observed (negative control). g–i) Fluorescently labeled (green, PKH67 dye) 3T3‐fibroblast‐derived EVs are only captured on CD63 antibody‐functionalized lipid patches (g) and not on EpCAM antibody‐functionalized lipid‐patches (h), or IgG1 isotype control antibody‐carrying lipid patches (negative control) (i). Scale bars equal 50 µm.

Clinical samples, such as blood samples, contain various EVs originating from different cell types, therefore, specific detection of tumor‐derived EVs is crucial for a robust diagnosis. To explore the specificity of our device, purified EVs from three different cell lines (MCF7, HT1080, and 3T3 fibroblast) were trialed. As a general EV biomarker, CD63 should be present on EVs released by any of the three cell lines.^[^ ^43^, ^44^, ^45^, ^46^, ^47^ ^]^ In contrast, only MCF7 (a breast cancer cell line of epithelial origin), but not HT1080 (a nonepithelial fibrosarcoma cell line) express EpCAM,^[^ ^48^ ^]^ as confirmed by fluorescence‐activated cell sorting (FACS) (Figure S3, Supporting Information). When incubating the respective EVs on AB‐functionalized lipid microarrays, the fluorescence signals reveal the described specificity (**Figure** **3**). All three types of EVs are captured on the CD63 functionalized lipid patches. In contrast, only the MCF7‐derived EVs are captured on EpCAM‐functionalized lipid patches. For the IgG1 isotype control AB none of the EV types is captured (as expected from the negative control). The results show, that by choice of adequate AB, the platform can specifically capture a certain type of EV.

### 2.4 Sensitivity of EV Capture

In order to investigate the sensitivity of our platform, EV dilution series were conducted for lipid microarrays functionalized with three different ABs (against CD9, CD63, and EpCAM, respectively). The concentration of MCF7‐derived EVs in nine purified samples from independently collected cell culture medium was quantified by ExoELISA to be (3.7 ± 2.2) × 10^10^ particles mL^−1^. The initial sample was diluted by a factor of 10 to obtain a starting point with ≈4 × 10^9^ EVs mL^−1^ for a dilution series in the physiological relevant range of EV abundance (10^3^–10^9^ mL^−1^) in patient blood.^[^ ^49^ ^]^ This sample range (4 × 10^9^ to 4 × 10^3^ EVs mL^−1^) was then incubated onto lipid arrays functionalized with different capture ABs.

**Figure 4:**
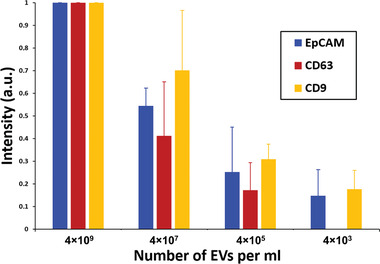
EV capture sensitivity. The graph shows the obtained fluorescence intensity on the lipid‐patch array functionalized with different antibodies (CD9, CD63, and EpCAM, respectively) for undiluted purified MCF7‐derived EVs (containing 4 × 10^9^ EVs per mL) and dilutions of 1:10^2^, 1:10^4^, and 1:10^6^, containing 4 × 10^7^, 4 × 10^5^, 4 × 10^3^ EVs per mL, respectively. The fluorescence intensity obtained with undiluted EVs is set to 1 for normalization. The error bars represent the SD from biological triplicates for each antibody.

The obtained fluorescence signals show that detection is still possible down to 1: 10^4^ dilution (translating to 5 × 10^5^ EVs mL^−1^) for CD63 ABs and even in a 1:10^6^ dilution (translating to 5 × 10^3^ EVs mL^−1^) for the EpCAM and CD9 ABs (**Figure** **4**), thus demonstrating the high sensitivity in the relevant EV concentration range. Additionally, the differences between the three ABs underline, that choice of an optimal AB is key for an efficient capture and maximal sensitivity.

### 2.5 Characterization of AB Immobilization

**Figure 5:**
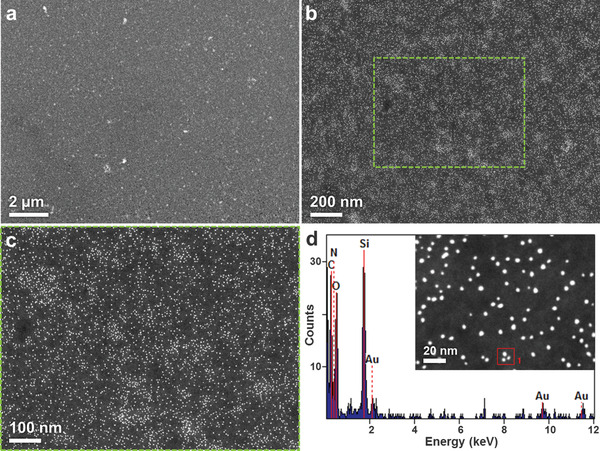
Antibody density on functionalized lipid patches. a,b) STEM images of an antibody‐functionalized lipid patch after incubation with Au‐NP conjugated secondary antibody. c) Close‐up of the area marked with the green box in (b). At this magnification, single Au‐NPs become discernable. d) EDX data on the particles within the red box of the inset, showing that the observed particles are indeed the Au‐NPs. Based on the observed Au‐NP density, 5.1 × 10^6^ antibodies are expected per 30 × 30 µm² lipid patch (translating to a density of ≈5.7 × 10^3^ µm^−1^).

To further characterize the platform, we strived to look at the immobilization density and role of the lipid membrane fluidity in the capture process. In order to investigate the average amount of immobilized ABs on a lipid patch, we used gold nanoparticle (Au‐NP)‐conjugated secondary ABs to detect the EV‐capture ABs bound to the lipid patches. The Au‐NPs were then visualized on the lipid patches by scanning transmission electron microscopy (STEM). The 2 nm Au‐NPs are clearly visible in the obtained images (**Figure** **5**).

As counting the particles on a whole 30 × 30 µm² lipid patch is not feasible, 10 smaller areas in two different magnifications and on different locations on a patch were scanned and the particles were identified and counted using ImageJ^[^ ^50^ ^]^ (Figure S4, Supporting Information). The size of one AB is around 10 nm,^[^ ^51^ ^]^ thus the Au‐NPs (2 nm) are significantly smaller and should not sterically affect the binding density. Although we cannot fully exclude the possibility of double conjugation, it seems reasonable to assume that there is a 1:1 ratio of primary AB to secondary AB and conjugated Au‐NP (or being at least of the same order). Following these assumptions, an average number of around 5.1 × 10^6^ ABs per 30 × 30 µm^2^ lipid patch, giving a density of 5.7 × 10^3^ µm^−1^. This is about a tenth of the number of accessible headgroups of a model allergen conjugated lipid in a lipid patch as estimated previously^[^ ^52^ ^]^ when adjusted to the same concentration. Keeping in mind that each streptavidin (as tetramer with a total of four biotin‐binding pockets, 2 each in opposite directions)^[^ ^53^, ^54^ ^]^ can potentially bind to biotinylated lipids at the membrane facing side, but only one of the bigger biotinylated ABs on the other side for steric reasons and that conjugation efficiency of the AB attachment to the lipid patch and the secondary conjugation with the Au‐NP carrying AB will both be not 100%, these results are still in reasonable agreement.

In addition to the previously discussed AB density, the role of attachment to the lipid layer in capture effectiveness is of interest. Various strategies for AB immobilization are discussed in the literature, in particular focusing on how to minimize the impact of linking strategy on AB function. While common chemical linking approaches, targeting amine‐, carboxyl‐, or thiol‐ groups usually work effortless, they cannot control the specific orientation of AB in relation to the surface, as these groups are available in abundance on the AB.^[^ ^55^, ^56^, ^57^ ^]^ Therefore, a fraction of the ABs will be oriented with their active site toward the surface, thus rendering them inactive for capture. Certain click‐chemistry approaches as well as making use of biochemical interactions (e.g., streptavidin‐biotin, protein A/G binding to ABs) can achieve an orientation‐controlled immobilization.^[^ ^56^ ^]^ However, these approaches are usually more elaborate, and, e.g., in the streptavidin‐biotin strategy, also the ABs need to be biotinylated site‐specifically, otherwise orientation is again random. All standard approaches have in common, that they bring the AB usually into very near vicinity to the surface with only a linker molecule in between. Therefore, it is of special interest, that it is reported in the literature, that anchoring of ABs to lipid membranes enhances the capture of circulating tumor cells (CTC)^[^ ^58^, ^59^ ^]^ and that tuning membrane fluidity of liposomes can be used to specifically target cancer cells.^[^ ^60^ ^]^ This points to a positive influence of such a “lipid cushion” on AB effectiveness in general. In contrast, commercial EV capture platforms like ExoView (Nanoview Biosciences, USA) and Exo‐Check (System Biosciences, USA) and approaches using plasmonic sensors directly immobilize antibodies on their devices.^[^ ^61^, ^62^, ^63^ ^]^ Therefore, we trialed our approach for the case of ABs directly bound to the solid substrate, leaving out the lipid‐patch cushion. To bind the ABs directly to a substrate, a click‐chemistry approach was used to generate surface bound biotin arrays as described previously.^[^ ^64^ ^]^ In short, biotin‐azide was spotted via microchannel cantilever spotting (μCS)^[^ ^65^ ^]^ onto dibenzocyclooctyne (DBCO) functionalized glass slides. Then biotinylated ABs were bound via streptavidin as linker resulting in arrays analogue to the ones described above, but without the lipid patches in between bound ABs and glass surface. This allows for a side‐by‐side comparison of directly substrate‐bound ABs (with only a short and rather rigid linker molecule between the glass and streptavidin/AB part of the sandwich) and the lipid‐patch‐based AB microarrays (with a full lipid membrane in fluid state between the streptavidin/AB and the supporting solid glass substrate). After incubating CD63 AB‐functionalized arrays of both types with fluorescently labeled EVs, the correlated fluorescence signal can only be detected on the lipid‐based microarrays, while the direct substrate‐bound sample only shows random attachment of EVs (Figure S5, Supporting Information). This striking difference can be accounted for by two reasons: (I) the mobility of lipid‐linked ABs within a lipid patch, and (II) denaturation of ABs in direct contact to the solid substrate. Orientation significantly affects the activity and efficiency of ABs and catalytic proteins.^[^ ^66^ ^]^ Here, the lipid‐membrane introduces a greater flexibility to the attachment, due to the membrane fluidity,^[^ ^26^, ^67^ ^]^ potentially mitigating accessibility problems. A similar effect was also reported in the capture of CTCs by lipid‐conjugated ABs, where the capture efficiency decreased significantly (from 82.1% to 63.5%) after fluidity of the utilized SLMs was reduced.^[^ ^59^ ^]^ Furthermore, the direct contact of the ABs with the solid surface can lead to denaturation and loss of function.^[^ ^68^ ^]^ The nonfouling properties of the lipid patches can minimize the impact on AB structure, as they interact much less strongly with the bound AB as a glass surface.

### 2.6 EV Capture from Unpurified Samples and EV Cargo Retention

EVs are abundant in bodily fluids and cell culture medium. Thus, samples can be easily obtained, but these liquids also contain a vast complex background of other components, such as lipids, lipoproteins resembling in size and density the EV proteins, and cell debris.^[^ ^23^, ^69^ ^]^ Therefore, most EV capturing approaches strictly require prepurification or pre‐enrichment steps, which are time‐consuming and expensive. Encouraged by the good performance of our platform in the previous tests, experiments with unpurified cell medium were conducted. Here, MCF7 conditional medium was only prefiltered with a 0.22 µm pore filter to remove cells or cell debris (>220 nm), which can be achieved in only 30 s. The filtered medium was then incubated on CD63 AB‐carrying lipid microarrays for EV capture without further purification or enrichment steps. Because the EVs were also not fluorescently labeled for these experiments, a fluorescent labeled EpCAM AB was used for immunostaining of the lipid patches after EV capture. With this general setup, a series of different experiments was conducted to evidence the reliable capture of EVs directly out of a complex media background by the lipid microarray platform.

**Figure 6:**
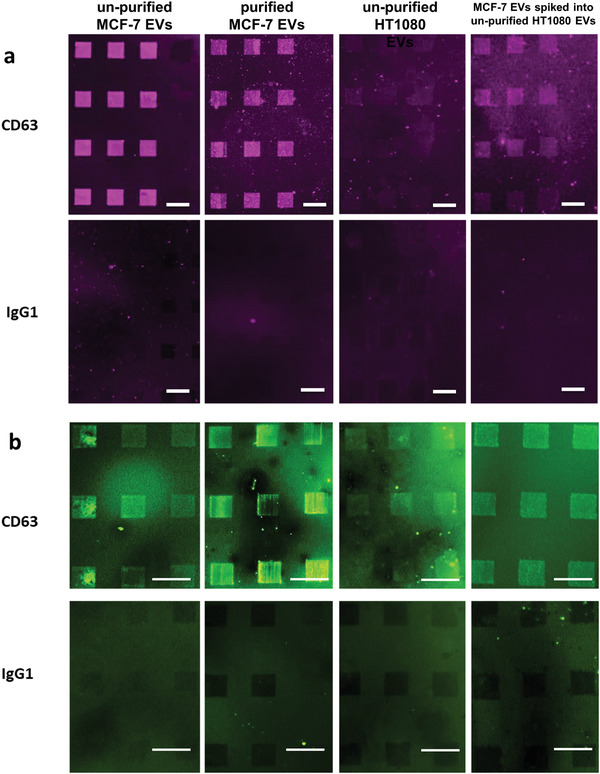
EV capture from complex samples and RNA retention. a) Fluorescence microscopy images of CD63 antibody‐ and IgG1 (isotype control) antibody‐functionalized lipid patches after capture of EVs from purified, unpurified and spiked samples and subsequent immunostaining against EpCAM (anti‐EpCAM antibody from rabbit, Alexa‐647 (in purple) conjugated anti‐rabbit antibody). MCF7‐derived EVs result in a strong fluorescence signal from purified and unpurified sample. Incubation of unpurified HT1080 derived EVs results (as expected) in only weak fluorescence. Incubation of HT1080 conditioning medium spiked with purified MCF7‐derived EVs also induces a fluorescence signal on the lipid‐patch array. The IgG1 (isotype control) carrying lipid patches remain without fluorescence for all cases (negative control). This shows that specific detection out of complex medium is possible, and the signal arises not from unspecific interactions with other components of the sample medium. b) Staining with a nucleic acid dye (SYTO in green) reveals that all CD63 antibody‐functionalized lipid arrays contain nucleic acids, while arrays carrying IgG1 (isotype control) antibodies do not. This shows, that RNA cargo present in the EVs remains in the lipid‐patch arrays after capture, thus could be used for downstream analysis. All scale bars equal 50 µm.

EV capture results showing a comparison of unpurified and purified samples are shown in **Figure** **6**. The unpurified MCF7 EVs show an even stronger EpCAM signal than the purified ones, probably because of damage and loss of EVs during the purifying steps. The HT1080 (as expected for being EpCAM negative) shows only a minimal fluorescent signal, caused by some remaining unspecific binding of the EpCAM‐AB used for secondary staining. To even further raise sample complexity and coming closer to a situation mimicking clinical samples, purified EVs of MCF7 were spiked into the conditional medium of HT1080 cells (500 µL of purified MCF7 EVs into 12 mL of the conditional medium of HT1080). This results in the breast‐cancer‐associated EVs being in a vast background of EVs and other biomaterials stemming from nonbreast‐cancer cells, thus getting closer to a real clinical sample in complexity. Still, the lipid microarrays show a clear signal of captured EpCAM‐positive EVs, thus indicating, that the MCF7 EVs could be successfully captured from the spiked sample. The reduced intensity can be understood as caused by the additional dilution and preparation steps during spiking and competitive binding of the HT1080 EVs and MCF7 EVs onto the CD63 AB carrying lipid microarrays. To further ensure that the interpretation of the binding experiments is correct and no unspecific interference of other components in the medium occurs, additional control experiments were implemented (Figure S6, Supporting Information).

Finally, the aspect of retaining the EV cargo in the system was examined, as the EV content is a rich source of diagnostic and scientific information.^[^ ^70^ ^]^ It was previously shown, that the content of EVs can be trapped on a surface when a SLM is formed by vesicle fusion of the EVs to the substrate.^[^ ^71^ ^]^ To elucidate whether the EV cargo is also retained on our platform, a RNA staining dye (SYTO RNASelect, only expressing green fluorescence when bound to RNA) was incubated onto the lipid microarrays after EV capture. Fluorescence signals were obtained from all lipid patches carrying CD63 AB (thus being able to capture EVs), while no fluorescence signal is detected on patches functionalized with IgG1 isotype control AB (Figure 6b). This indicates, that the RNA in the EVs bound to the lipid patches remains trapped in our system, opening up a future downstream analysis, e.g., by targeted retrieval of patches via micropipettes, as previously shown for CTCs.^[^ ^72^, ^73^ ^]^

### 2.7 EV Capture from Patient Sample

**Figure 7:**
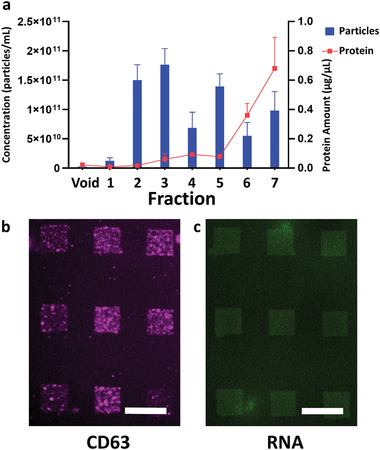
EV capture from patient samples. a) NTA/BCA data on the different fractions obtained from 3 cancer patients. The error bars report the SD. b) Fluorescence microscopy images of CD63 antibody‐functionalized lipid patches after capture of EVs from fraction 5 of patient I and subsequent immunostaining against EpCAM (anti‐EpCAM antibody from rabbit, Alexa‐647 (in purple) conjugated anti‐rabbit antibody) and c) staining for nucleic acid (SYTO in green) confirming RNA cargo retention. Scale bars equal 50 µm.

To validate our platform further we strived to demonstrate detection from EVs isolated from blood samples of cancer patients. For this, serum was collected from three pancreatic cancer patients and EVs were enriched using SEC (qEV35 nm columns). All fractions were characterized by NTA; BCA and then incubated on lipid‐patch arrays functionalized with CD63 AB. Successful capture of EVs was confirmed by subsequent staining with EpCAM AB, recognizing cancer‐derived EVs^[^ ^19^ ^]^ (**Figure** **7**b).

In all three patients, EVs could successfully be detected on the CD63 functionalized patches by EpCAM immunostaining, while no signal was obtained from negative control fractions (Figure S7, Supporting Information). To test if EV nucleic acid cargo is retained on the lipid array, we stained for RNA (Figure 7b; and Figure S8, Supporting Information). The staining is supporting the assumption of EV cargo being retained, further corroborating the data on RNA retention in the previous section. Overall, the successful capture of cancer‐associated EVs from the patient samples underlines the potential of our platform for clinical applications.

## 3 Conclusion

Despite the large variety of new isolating or detecting methods that try to conquer the challenges of EV isolation or detection,^[^ ^10^ ^]^ there is still no approved EV isolation, or detection method introduced for clinical use. This is partly because of the high standard that such a system would need to fulfill, as it should be accurate, specific, reliable, rapid, easy to handle, inexpensive, and applicable for different types of EVs.^[^ ^24^ ^]^ Toward this goal, we presented our new approach of utilizing dip‐pen nanolithography created lipid patches arranged into microscale arrays for EV capture. The platform can detect EVs from purified and unpurified samples rapidly (overall processing times of 2 h instead of standard processes that require up to 17 h) and works with minimal sample volumes (30–50 µL), minimizing costs for processing and material consumption. It was shown that the platform can reliably target EVs highly accurate and specific, out of a background of complex media and in mixtures containing off‐target types of EVs. The obtained sensitivity is matching that of other ultrasensitive methods (comparison to selected EV capture methods in Table S1, Supporting Information),^[^ ^74^, ^75^, ^76^, ^77^, ^78^, ^79^, ^80^, ^81^ ^]^ spanning the whole range of physiological relevant EV concentrations. Furthermore, the lipid arrays were also demonstrated to retain the RNA (and thus probably the other biomaterial cargo) of EVs in the system, which shows the platforms potential to isolate RNA for downstream analysis. Finally, the detection of cancer‐associated EVs from pancreatic cancer patients validates our platforms robustness and potential for clinical application. The ABs used in the platform can be readily exchanged for targeting different types of EVs without need for adjustments at the general approach, enabling a high versatility. By incorporation of the platform into microfluidic chips, even easier handling could be achieved.^[^ ^82^ ^]^ In combination with full‐fledged lab‐on‐chip approaches, the lipid‐array‐based EV capture could be used in point‐of‐care or even at bedside environment.^[^ ^83^, ^84^, ^85^ ^]^ In conclusion, the presented novel EV capture platform offers a high potential for diagnostic and research applications as demonstrated for, but not limited to cancer, as many other applications involving EVs could be addressed, alike.

## 4 Experimental Section

### Phospholipids

20 mg mL^−1^ (25.4 × 10^−3^ m) of 1,2‐dioleoyl‐*sn*‐glycero‐3‐phosphocholine (DOPC; Avanti Polar Lipids, USA) and 5 mol% of 1,2‐dioleoyl‐*sn*‐glycero‐3‐phosphoethanolamine‐*N*‐(cap biotinyl) (Biotin‐PE; Avanti Polar Lipids, USA) in DOPC were used in patterning the lipid patches. All lipids were purchased from Avanti Polar Lipids, USA.

### Patterning of Lipid Arrays by DPN

The lipid micropattern patches were fabricated by DPN 5000 (Nanoink Inc., USA) with the B‐side of M‐type cantilever array which has 12 tips with a 66 µm pitch (Advanced Creative Solutions Technology (ACST, USA). Tips were homogenously coated by dipping into the matched inkwells (ACST, USA) for 5 min with a controlled 60% relative humidity (RH). The inkwell was loaded with 2 µL of the desired phospholipid mixture (20 mg mL^−1^ in chloroform) in each reservoir and chloroform evaporated in a vacuum desiccator (15 min) or overnight in a damp‐proof box. Afterward, the coated tips were moved to the desired location on the substrate (either glass cover slips cleaned by sonification subsequently with chloroform, isopropanol and DI water, or 2‐methacryloyloxyethyl phosphorylcholine (MPC) copolymer covered slides^[^ ^31^ ^]^) and lipid‐patch arrays were written. The patches (30 × 30 µm^2^) were written at 30–40% RH with hatch lines of 0.5 µm pitch and a writing speed of 0.2–2 µm s^−1^.

### Purification of Extracellular Vesicles

MCF7 cells, HT1080 cells, and 3T3 fibroblasts were cultured in Dulbecco's modified Eagle's medium (DMEM, Life Technologies, Germany) supplemented with 10% exosome‐depleted FBS (System Biosciences, USA) under standard cell‐culture conditions (37 °C and 5% CO_2_) for 48 h. Then, condition medium was centrifuged at 500*g* for 10 min to remove suspended cells. The condition medium was then filtered by a 0.22 µm Steriflip filter (Millipore, USA) on ice for removing cell debris. In order to do further enrichment of EVs and reduce the amount of the condition medium, it was centrifuged with Amicon 100 kDa cut‐off Ultrafilter (Millipore, USA) at 5000*g* for 30 min. The filtered medium was discarded and the EVs were fully recovered by using 200 mL of cold phosphate buffered saline (PBS) into the filter device to sweep the filter membranes. The enriched EVs sample was stained with the PKH dye (Sigma‐Aldrich, USA), which is a lipophilic fluorescent dye, and then centrifuged again at 5000*g* for 30 min for removing unbounding dyes. All centrifuge and filtering steps were performed at 4 °C. The labeled EVs were fully recovered from the spin column and loaded into a qEVoriginal SEC column (IZON, UK). Fractions of 500 µL each were collected according to the recommendations of the supplier (meaning that the first 3 mL void volume was discarded as the EV fraction is expected to appear at (3.75 ± 0.25) mL. “Fraction 1” corresponds to the first 500 µL sample collected after the void volume passed the column. Additional fractions refer to the subsequent volumes of 500 µL passing, respectively. SEC was done at room temperature, collected fractions were immediately transferred on ice or stored at −80 °C after collection.

### Micro BCA Analysis

150 µL of each standard and fractions were loaded into a microplate's well and replicated twice. Each well was added with 150 µL of the working reagent and mixed thoroughly on a plate shaker for 30 s. The loaded microplate was then incubated for 2 h at 37 °C. The result was measured for absorbance at 562 nm on a plate reader.

### Nanoparticle Tracking Analysis (NTA)

EV concentration and size distribution were determined by ZetaView (Particle Metrix, Germany). Samples were diluted consecutively with ddH_2_0 to receive an optimal concentration of 100–200 particle counts. One mL of diluted sample was injected into the NTA. The size distribution was measured in scatter mode and Zeta potential was measured in Zeta Potential mode using set up: Sensitivity 85; Min size 20 nm; Max size 1000 nm; Min Brightness 10. The ZetaView determines the size of vesicles based on Brownian motion and this principle is used for analysis of nanometer‐sized particles.^[^ ^86^ ^]^

### Vesicles Labeling with PKH Dyes

After the ultrafilter, Diluent C buffer (Sigma‐Aldrich, USA) was added into condensed medium (total volume 1 mL) and then mixed well with 1 mL of labeling solution (consisting of 6 µL of the PKH67 or PKH26 ethanolic dye (as obtained from Sigma‐Aldrich, USA) in 1 mL Diluent C). The mixture was incubated for 5 min at room temperature and to stop the staining reaction, 1% BSA (Sigma‐Aldrich, USA) in PBS buffer (2 mL) was added for 1 min.

### EV Chip Functionalization and Capturing

The micropattern lipid patches on EV chip were coated by 1% streptavidin (Sigma‐Aldrich, USA) in 10% BSA (Sigma‐Aldrich, USA) in PBS for 30 min and washed 3 times by pipetting on and off 100 µL of PBS to wash away unbound streptavidin. The streptavidin‐lipid patches were then incubated with 100 µL of 0.01 µg µL^−1^ solution of desired biotinylated antibody ((Biotinylated anti‐EpCAM (VU‐1D9, abcam, UK), biotinylated anti‐CD63 (MEM‐259, abcam, UK), biotinylated anti‐CD63 (Rabbit antimouse; MyBioSource, USA) and biotinylated Mouse‐IgG1 isotype control (MOPC‐21, abcam, UK)) in 10% BSA in PBS for 30 min and washed 3 times by pipetting on and off 100 µL of PBS, rendering the EV chip ready to use. The purified EVs were diluted to 10^2^–10^8^ times with 4% EDTA‐free Protease Inhibitor cocktail (Sigma‐Aldrich, USA) in cold PBS. The purified and diluted EVs fraction (50 µL) was loaded onto the prepared EV chip for 1 h at 4 °C. After incubation, the sample was washed by dipping it into 100 mL of DI water for 3 times. Samples where then imaged with a fluorescence microscope.

### EV Dilution Series

For the dilution series experiments, MCF7 EVs were purified as described above and EV concentration was determined by ExoELISA (see next section). Then the EV samples were diluted to the respective concentration and incubated on lipid patch arrays functionalized with the respective ABs as described above. After EV capture, the samples were stained and analyzed by fluorescence microscopy. Fluorescence intensities for 5 patches on each array were quantified with the onboard software of the microscope (NIS‐Elements, Nikon, Germany). For each AB the experiment was done in triplicate with independently prepared EV samples.

### ExoELISA

The ExoELISA‐Ultra CD63 kit (System Biosciences, USA) was used for calculating EV concentration for the purified samples used in the dilution series. 50 µL of EV samples and standards were loaded into microtiter plate and incubated at 37 °C for 1 h. After incubation, the reacted wells were washed 3 times by 100 µL of 1× wash buffer for each well. 50 µL of diluted CD63 primary antibody (1:100) in blocking buffer was added to each reacted well and incubated at room temperature for 1 h with shaking (parameter). Before adding 50 µL of 3,3”,5,5”‐tetramethylbenzidine (TMB) substrate to each reacted well, washing step with 1× wash buffer was repeated. After incubating with TMB substrate at room temperature for 5–15 min with shaking, 50 µL of stop buffer was loaded to each well and read immediately by an ELISA reader at 450 nm wavelength.

### Capture of EVs from Unpurified Condition Medium

After incubating 48 h, the condition medium was centrifuged at 500*g* for 10 min in order to get rid of the big projects (suspensive cells, cell debris, etc.) and subsequently filtered by 0.22 µm Steriflip filter on ice. The prepared EV chip was directly immersed in the filtered medium in a petri dish (VWR, USA) at 4 °C for 1 h. The chip was washed three times by dipping into 100 mL DI water after incubation and imagined with a fluorescence microscope.

### Spiking Experiment

The MCF7 EVs were purified by SEC column (qEVoriginal, IZON LtD) as described above. 500 µL of purified MCF7 EVs was spiked into 12 mL of the condition medium of HT1080 which was cultured for 48 h. The condition medium of HT1080 was centrifuged at 500*g* for 10 min and filtered by 0.22 µm PES filter (Millipore, USA) in advance. The purified MCF7 EVs and HT1080 condition medium were mixed well and poured onto functionalized EV chips placed in a petri dish on ice for 1 h. After capturing, the EV chips were washed with DI water (three times by dipping into 100 mL) and then analyzed via optical microscopy.

### Immunostaining

0.5 µg of primary anti‐EpCAM antibody (Rabbit polyclonal, Abcam, UK) admixed with 50 µL of 10% BSA in PBS was incubated on the captured EV chip at 4 °C for 1 h and washed three times by dipping into 100 mL of DI water. Then, 0.5 µg of secondary anti‐rabbit IgG Alexa Fluor 647 (donkey polyclonal, Abcam, UK) mixed with 50 µL of 10% BSA in PBS was incubated at 4 °C for 1 h and then washed three times by dipping into 100 mL DI water, and imagined with a fluorescence microscope.

### Fluorescent Labeling of RNA

SYTO RNASelect Green Fluorescent Cell Stain (5 × 10^−3^ m, Thermo Fisher Scientific, USA) was first diluted to 5 × 10^−9^ m into PBS then immediately incubated onto captured‐EV chips for 20 min at room temperature. After incubation, the EV chips were washed by incubation with 100 µL of DI water two times for 5 min each. Then, the EV chips were imagined with fluorescent microscopy.

### Detection of Cancer‐Associated EVs from Patient Samples

Serum samples were collected from pancreatic cancer patients with approval of the local ethics committee at the University of Freiburg following informed consent from the donors. The serum samples were centrifuged at 1650*g* for 15 min according to the recommendations of the vacutainer supplier. The platelets‐free serum was transferred into fresh tubes and frozen at –80 °C. 500 µL of cell‐free serum were subjected to SEC, using qEV35 original columns (IZON, France). For each patient, 7 subsequent fractions (Fraction 1–7) of 500 µL each were collected after the void volume of 3 mL has passed the column. Also 500 µL of the void volume was sampled (Void) as control. 80 µL of each fraction was loaded onto prepared EV chips and incubated for 1 h at 4 °C. The EV chip was washed three times by dipping into 100 mL of DI water after incubation and then immunostained as described above. For two patient samples, RNA staining was performed as described above. After staining procedures, samples were imaged with fluorescence microscopy.

### Optical Microscopy

The images were recorded using an Upright microscope (Eclipse 80i, Nikon Instruments Europe B.V., Germany) using onboard software (NIS‐Elements, Nikon, Germany). Illumination for fluorescence microscopy was an Intensilight (Nikon, Germany) and filter cubes for TexasRed (excitation/emission wavelength: 559/630 nm, color‐coded red) FITC (475/530 nm, color‐coded green), and Cy5 (604/712 nm, color‐coded purple) were used.

### Electron Microscopy

A FEI Titan 80–300 aberration (image) corrected transmission electron microscope (TEM) operating at 300 KV acceleration voltage and equipped with a high‐angle annular dark‐field (HAADF) detector (Fischione) for STEM imaging, and a S‐UTW EDX detector (EDAX Inc.). TEM samples were prepared by direct printing. The lipid micropattern patches were directly fabricated on a silicon nitride (SiN) 20 nm thick membrane TEM window grid with 9 electron‐transparent windows, 8 of dimensions 100 × 100 µm^2^, and one of 100 × 350 µm^2^, by writing a lipid mixture of DOPC and Biotin‐Cap‐PE with a DPN 5000 system (Nanoink Inc., USA). The details of printing condition can be seen above section (Patterning of lipid arrays by DPN). The patterns on the SiN grid were coated 1% streptavidin and then incubated with 0.01 µg µL^−1^ of primary biotinylated‐antibody from rabbit (Abcam, UK). After washing, the primary antibody‐lipid arrays were incubated with secondary antibody conjugated with 2 nm gold particles (Abcam, UK) for 1 h at room temperature. Then, unbound secondary antibodies were washed away by pipetting on and off with DI water three times. The lipid arrays were ready for imaging by STEM. Au NPs were counted using ImageJ software where Intermodes threshold in the multithreshold option with a median filter of 2.0 pixel were used to eliminate the background noise before counting the nanoparticles at different magnifications (Figure S4, Supporting Information).

## Conflict of Interest

H.L., H.F., I.N., and M.H. submitted a joint patent application regarding the lipid‐patch‐based EV capture.

## Supporting information

Supporting Information

## Data Availability

The data that support the findings of this study are available from the corresponding author upon reasonable request.
