# Supplementary material for: Rapid Capture of Cancer Extracellular Vesicles by Lipid Patch Microarrays
Source: Adv Mater. 2021 Jul 26;33(35):2008493. doi: 10.1002/adma.202008493 (PMC11468818; doi:10.1002/adma.202008493)
Supplement: Supplementary file 1 — Supporting Information [file ADMA-33-2008493-s001.pdf]

© 2021 Wiley-VCH GmbH

ADVANCED MATERIALS

# Supporting Information

for *Adv. Mater.,* DOI: 10.1002/adma.202008493

# Rapid Capture of Cancer Extracellular Vesicles by Lipid Patch Microarrays

*Hui-Yu Liu, Ravi Kumar, Chunting Zhong, Saleh Gorji, Liliia Paniushkina, Ramsha Masood, Uwe A. Wittel, Harald Fuchs, Irina Nazarenko, and Michael Hirtz**

# Supporting Information

**Rapid Capture of Cancer Extracellular Vesicles by Lipid Patch Microarrays**

*Hui-Yu Liu, Ravi Kumar, Chunting Zhong, Saleh Gorji, Liliia Paniushkina, Ramsha Masood, Uwe A. Wittel, Harald Fuchs, Irina Nazarenko, and Michael Hirtz**

Dr. H.-Y. Liu, Dr. R. Kumar, C. Zhong, S. Gorji, Prof. H. Fuchs, Dr. M. Hirtz
Institute of Nanotechnology (INT) & Karlsruhe Nano Micro Facility (KNMF), Karlsruhe Institute of Technology (KIT), Hermann-von-Helmholtz-Platz 1, 76344 Eggenstein-Leopoldshafen, Germany
E-mail: michael.hirtz@kit.edu

S. Gorji
Joint Research Laboratory Nanomaterials (KIT and TUD) at Technische Universität Darmstadt (TUD), Jovanka-Bontschits-Str. 2, 64287 Darmstadt, Germany

Prof. H. Fuchs
Physikalisches Institut & Center for Nanotechnology (CeNTech), Westfälische Wilhelms-Universität, Wilhelm-Klemm-Straße 10, 48149 Münster, Germany

L. Paniushkina, R. Masood, Prof. I. Nazarenko
Institute for Infection Prevention and Hospital Epidemiology, Medical Centre, Faculty of Medicine, University of Freiburg, Freiburg, Germany

Prof. I. Nazarenko
German Cancer Consortium (DKTK), Partner Site Freiburg and German Cancer Research Center (DKFZ), Heidelberg, Germany

Prof. U. A. Wittel
Department of General and Visceral Surgery, Centre of Surgery, Medical Centre, Faculty of Medicine, University of Freiburg, Freiburg, Germany.

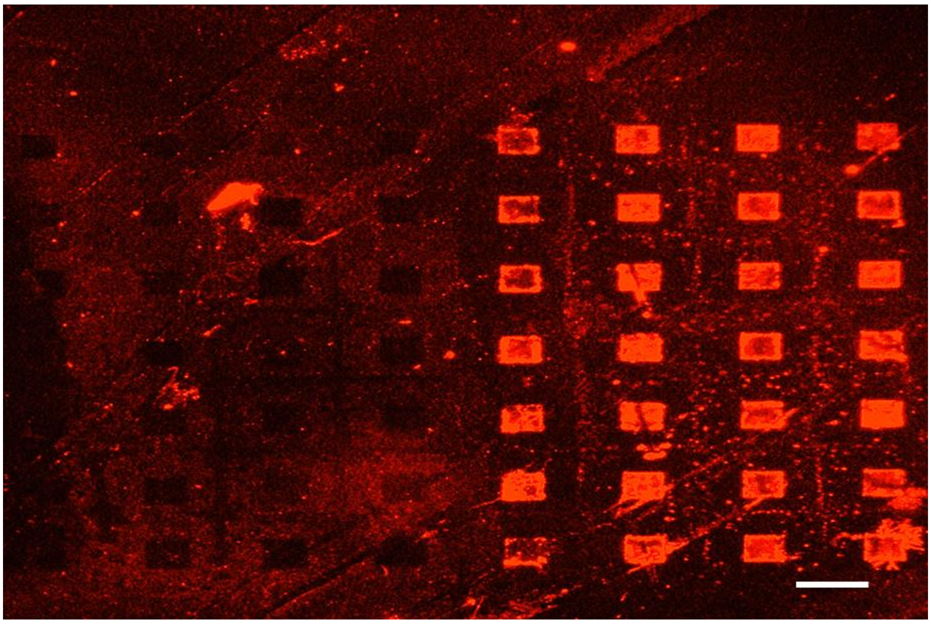

**Figure S1.** Control experiment for unspecific PKH dye adhesion. Fluorescent microscopy image of a lipid microarray after incubation with PKH26 stained MCF7 EVs. Only the EpCAM antibody-functionalized array columns on the right light up in fluorescence, while the non-functionalized (DOPC only) columns on the left remain dark. This indicates that no unspecific adhesion of PKH dye or unspecific EV fusion with non-functionalized lipid patches occurs. The non-functionalized patches even appear darker compared to the substrate background, indicating the non-fouling properties of DOPC which even suppresses unspecific adhesion compared to the naked substrate. Scale bar equals 50 µm.

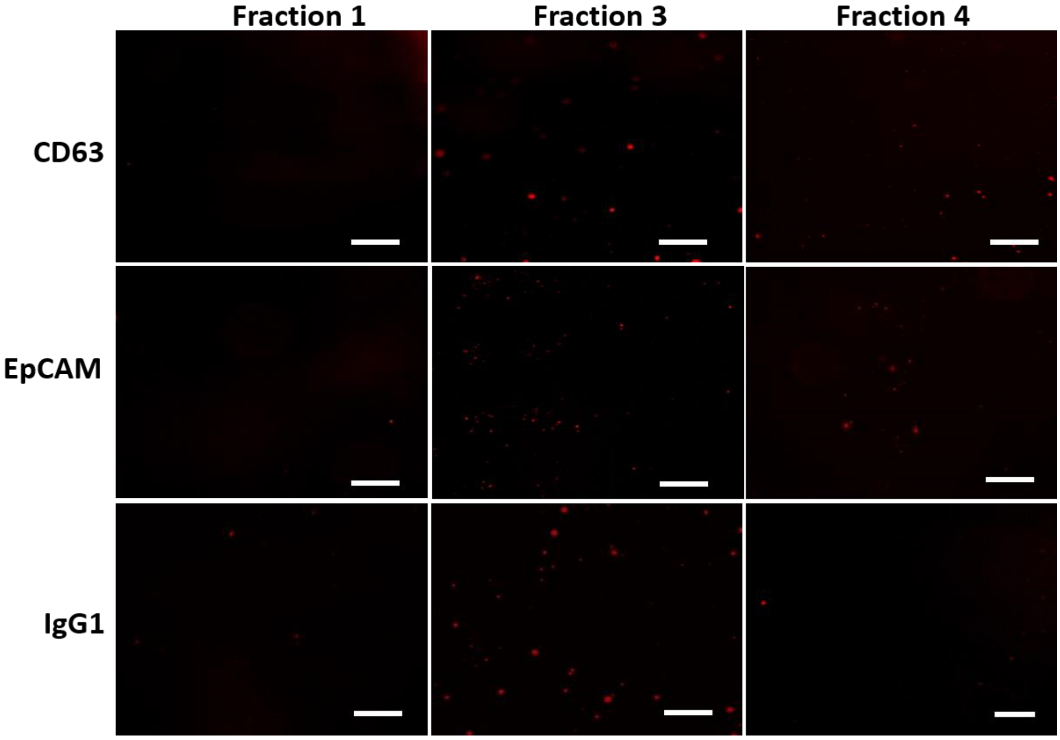


**Figure S2.** Control experiment with other purification fractions. Incubation of the other (non-EV) fractions of the purification process on AB functionalized lipid microarrays does not lead to any significant fluorescence signals from the lipid patches. Scale bars equal 50 µm.

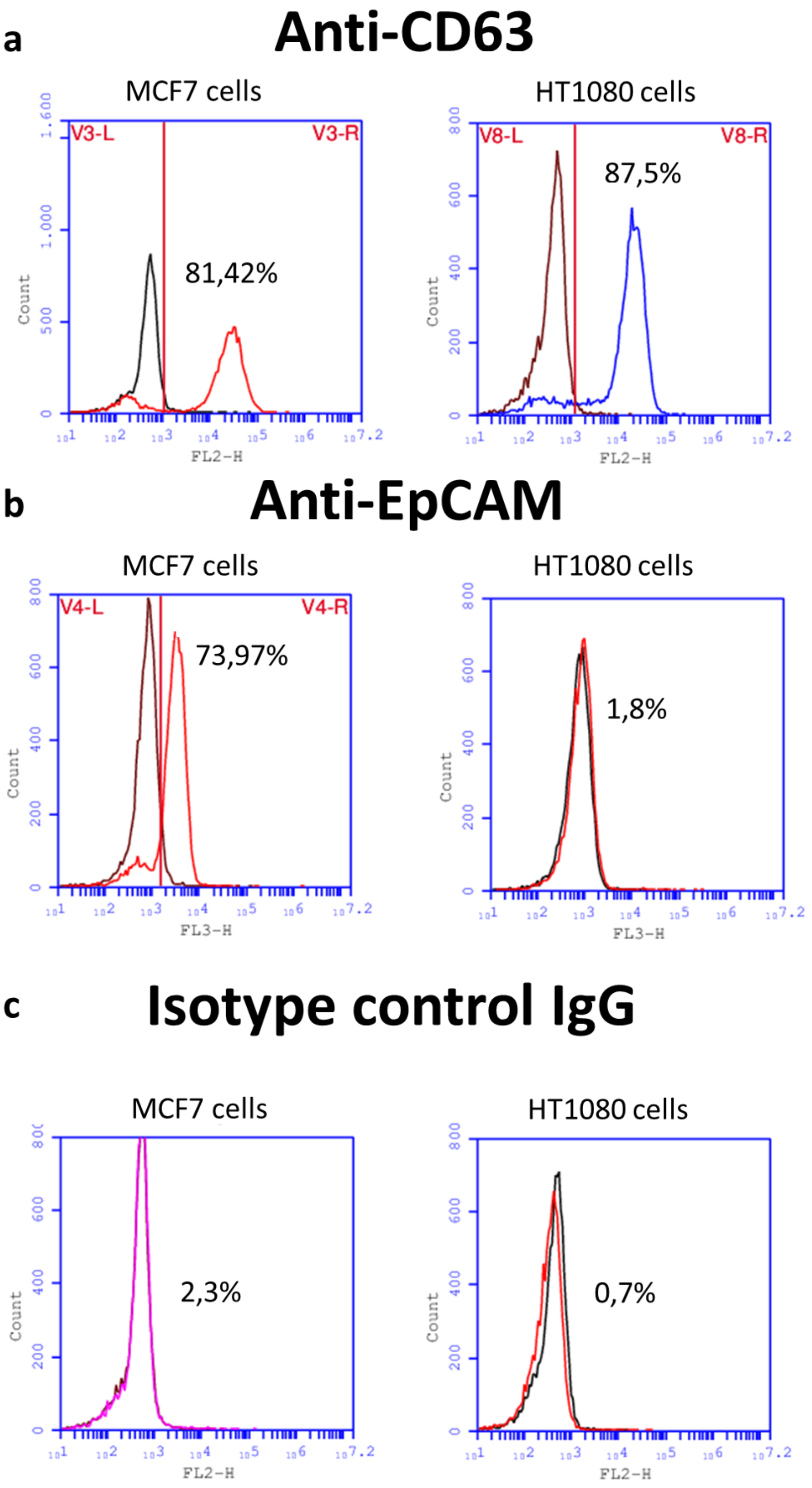


**Figure S3.** FACS analysis of MCF7 and HT1080 cells. a) Both cell lines are CD63 positive. b) Only MCF7 cells are strongly EpCAM positive with only minor interaction in HT1080. c) Both cell lines show negligible interaction with the isotype control IgG.

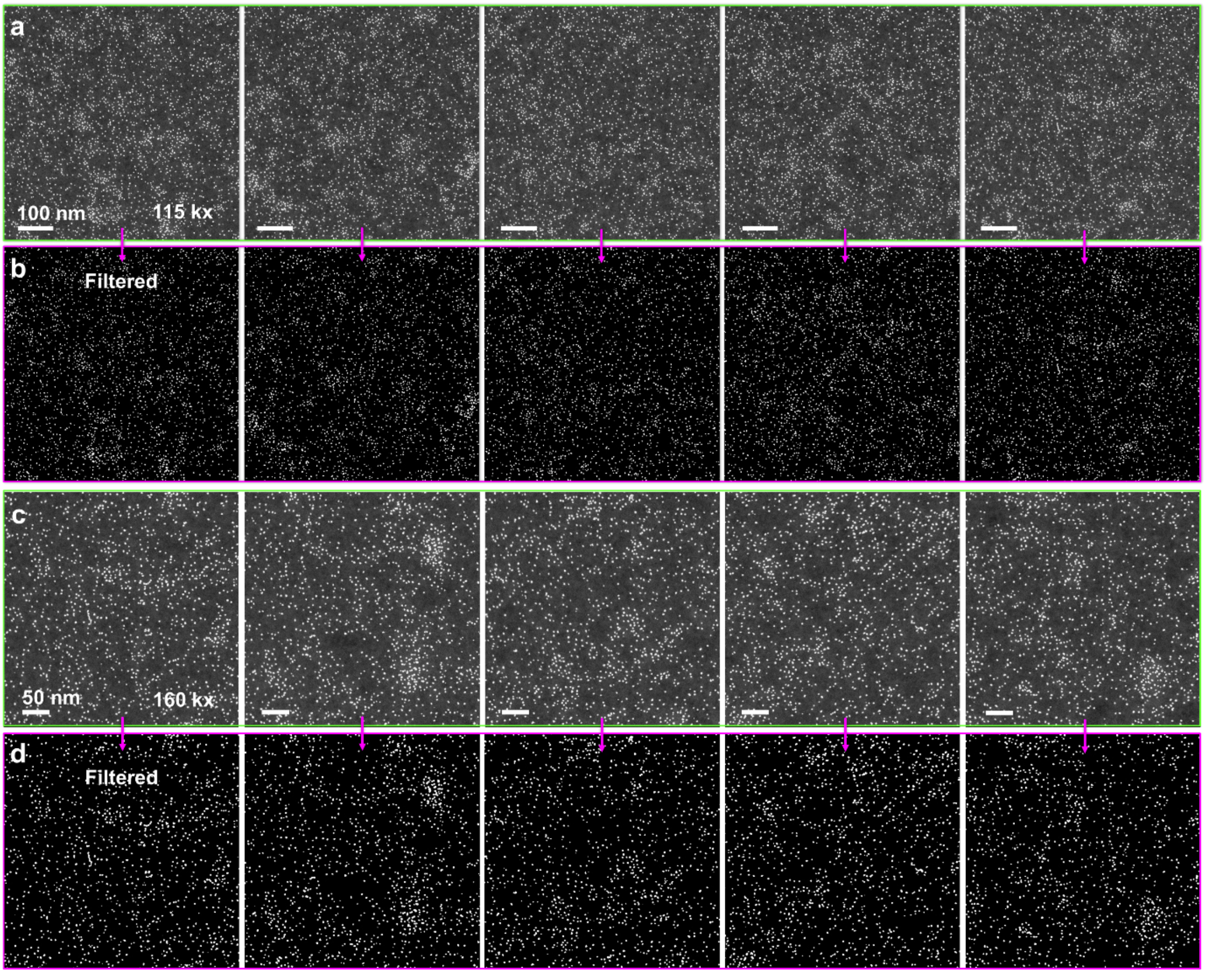


**Figure S4.** STEM images of Au-NPs bound to a lipid patch. a) The original STEM images (magnification of 115.000×, scale bars equal 100 nm) of the 2 nm sized Au-NPs on the lipid patch. b) Pictures after identification of Au-NPs by ImageJ. The area of each image is 666 × 666 nm² or 0.443 µm². The average number of Au-NPs in each image is 2530 ± 101. Extrapolating from these images, around $5.712 \times 10^3 \pm 228$ Au-NPs are bound per µm². This translates to $\sim 5.1 \times 10^6 \pm 0.2 \times 10^6$ ABs on a lipid patch of 30 × 30 µm². As consistency check, the analysis was repeated with c) even higher magnification images (160.000×, scale bars equal 50 nm), d) thresholded in the same way. Here, 1269 ± 35 particles are counted per area of 471 × 471 nm², yielding $5.723 \times 10^3 \pm 159$ Au-NPs bound per µm². Extrapolating onto a whole patch, $\sim 5.1 \times 10^6 \pm 0.2 \times 10^6$ ABs are expected, in agreement with the analysis of the lower magnification images.

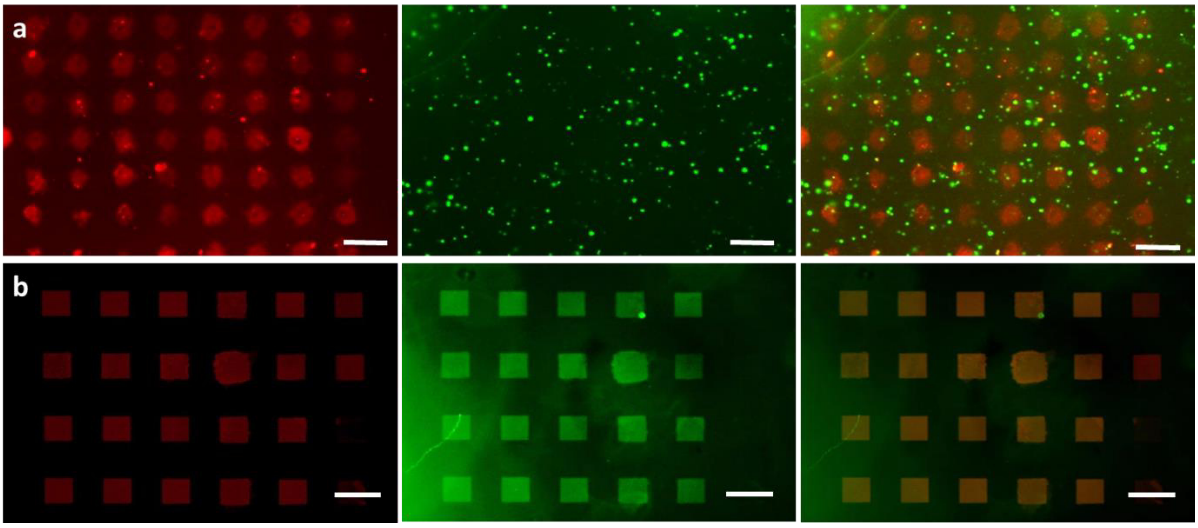


**Figure S5.** Direct surface immobilization vs. lipid patch based immobilization. a) Fluorescent image of a click-chemistry bound CD63 AB microarray after incubation with fluorescently labelled MCF7 EVs (PKH67 dye, green fluorescent). To visualize also the microarray itself, fluorescently labelled streptavidin (streptavidin-Cy3, red fluorescent) was used for building up the AB sandwich structure. While the microarray it-self is clearly visible (left), only random attachment of EVs is observed (middle) with no correlation between array features and EV attachment visible in the overlay (right). b) The parallel experiment on the lipid microarray bound ABs shows a perfect correlation between the lipid microarray (left), captured EVs (middle) in the overlay image (right). Scale bars equal 50 µm.

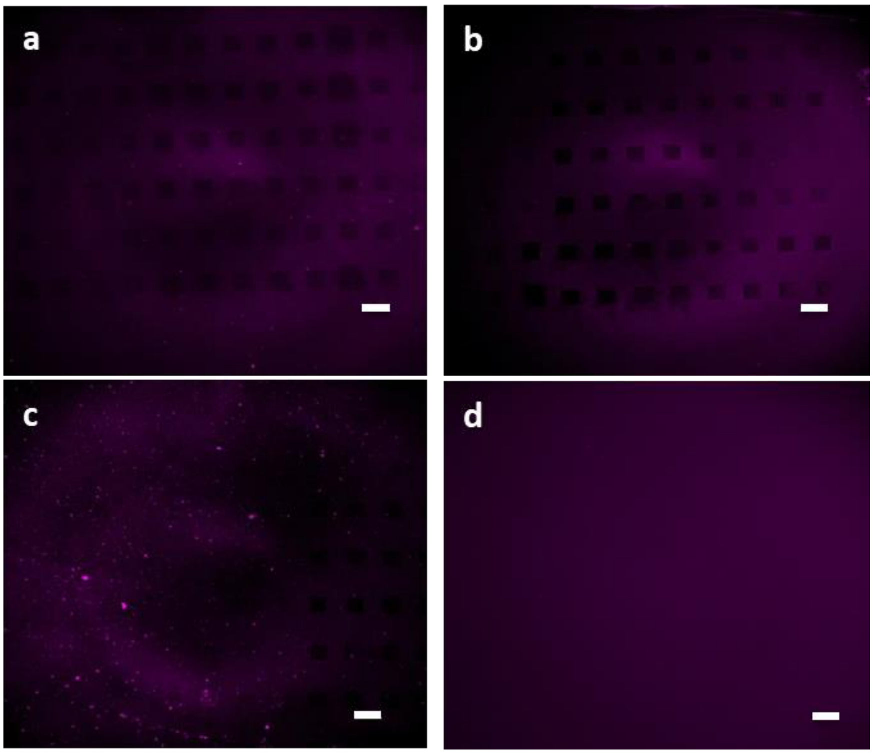


**Figure S6.** Control experiments for EV capture from unpurified conditional medium. a) To exclude unspecific binding of EVs or other interfering components of the conditional medium to the lipid microarrays were coated only with streptavidin, not carrying any anti-CD63 AB. After incubation with MCF7 EVs from unpurified conditional medium and subsequent staining against EpCAM (rabbit anti-EpCAM AB, Alexa-647 conjugated anti-rabbit secondary AB), no signal rises on the lipid patches, indicating a successful negative control (no unspecific binding). b) To exclude unspecific interactions of the secondary detection AB, lipid microarrays with anti-CD63 AB were incubated with MCF7 EVs as before, but then directly incubated with the secondary detection AB (Alexa-647 conjugated anti-rabbit AB) without prior incubation with the primary detection AB (rabbit anti-EpCAM AB). No signal is visible on the lipid patches, showing that the secondary detection AB has no significant unspecific interaction with the lipid microarrays and EVs. c) Lipid microarrays carrying IgG1 isotype control AB show no detection signal after MCF7 EV incubation and subsequent staining, indicating no unspecific interaction of EVs and the lipid microarrays. d) When anti-CD63 AB carrying lipid microarrays, after incubation with MCF7 EVs, are incubated with rabbit IgG isotype control AB (instead of rabbit anti-EpCAM detection AB), subsequent incubation with the fluorescently labelled anti-rabbit secondary AB also induced no signal. This indicates that there is also no unspecific interaction between non-target detection ABs and the lipid microarrays or the bound EVs. The scale bars in all images equal 50 µm.

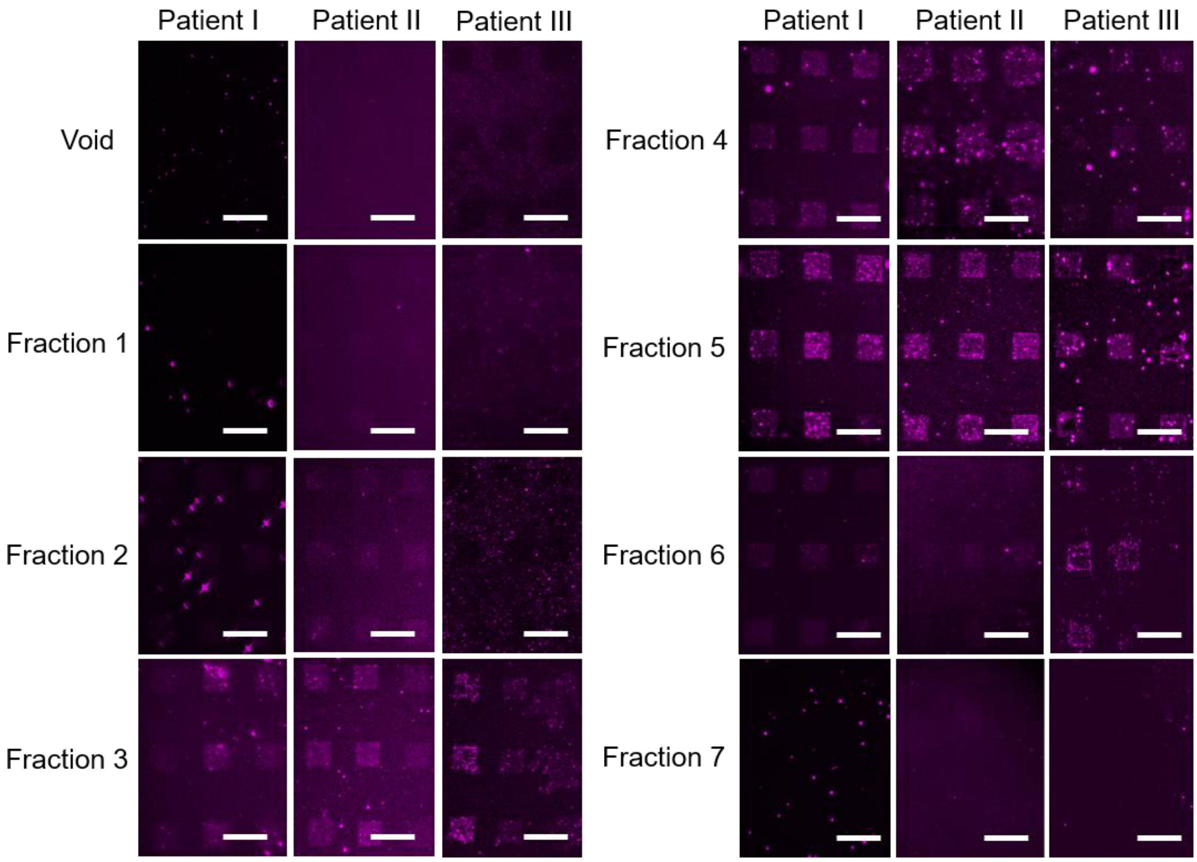


**Figure S7.** EV capture from patient samples. The image shows CD63 antibody-functionalized lipid patches after EV capture from patient sera and staining against EpCAM (rabbit anti-EpCAM AB, Alexa-647 conjugated anti-rabbit secondary AB). The columns correspond to the different patients, the lines then give the results for different fractions and the negative control (Void). All scale bars equal 50 µm.

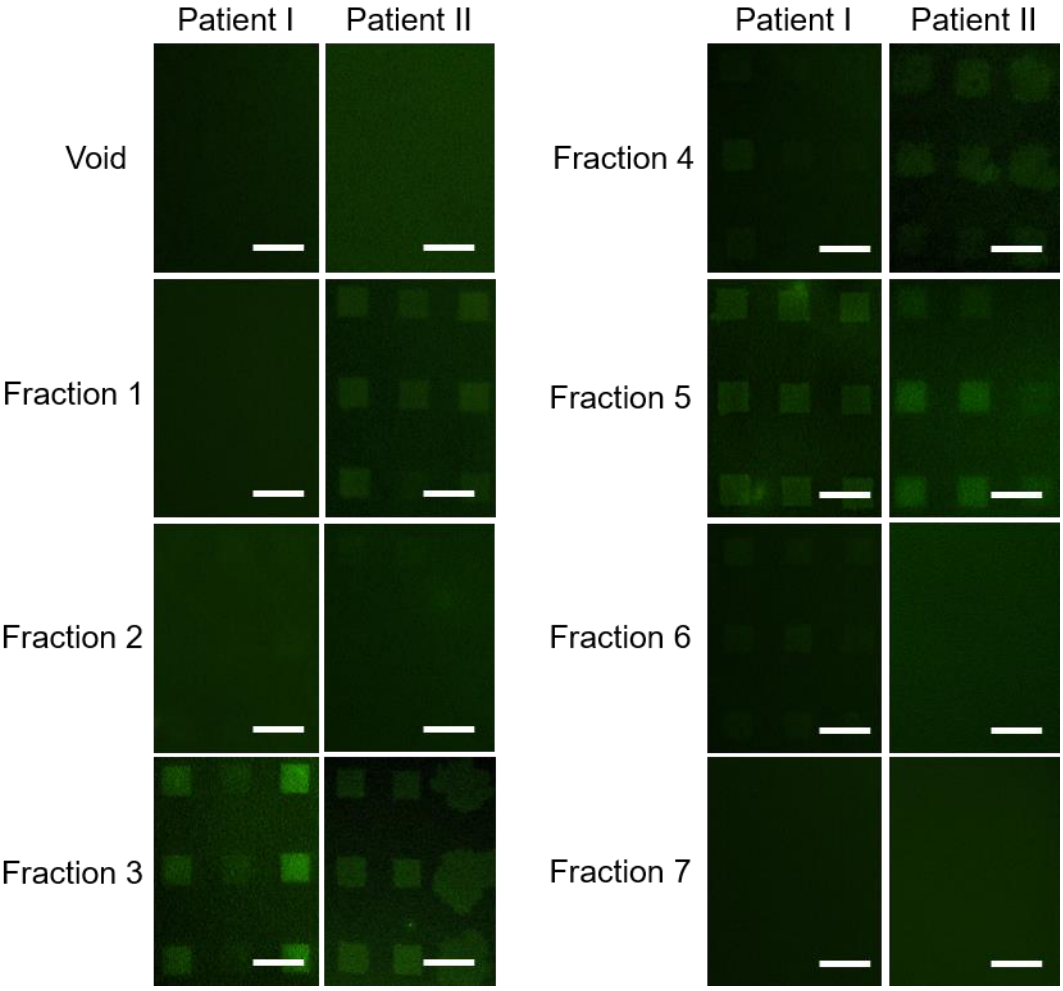


**Figure S8.** RNA retention from patient samples. For two of the patients, additional staining for RNA detection was performed. The image shows CD63 antibody-functionalized lipid patches after EV capture from patient sera and staining for nucleic acid with SYTO confirming RNA cargo retention. The columns correspond to the different patients, the lines then give the results for different fractions and the negative control (Void). All scale bars equal 50 µm.

**Table S1.** Comparison of EV studies.

| | Kanwar et al. [1] | Zhang et al. [2] | Zhao et al. [3] | Reátegui et al. [4] | Xu et al. [5] | Chen et al [6] | Zhang et al. [7] | Wang et al. [8] | This work |
|---|---|---|---|---|---|---|---|---|---|
| **Capture Approach** | Flat PDMS channel functionalized with specific capture ABs | Graphene oxide/ polydopamine nano-interface functionalized with specific capture ABs | Magnetic beads functionalized with specific capture ABs, immobilization by magnet | Herringbone microstructures functionalized with specific capture ABs | Magnetic beads functionalized with specific capture antibody, immobilization by magnet | Magnetic beads functionalized with specific capture antibody, immobilization by magnet | Nanoporous herringbone microstructures functionalized with specific capture ABS | Magnetic beads functionalized with specific capture antibody, immobilization by magnet | Arrays of supported lipid membranes functionalized with specific capture antibody |
| **Detection Method** | Membrane staining dye, fluorescence platereader | Secondary AB fluorescence / ELISA | Secondary AB fluorescence | Secondary AB fluorescence / RNA detection in lysate | Electrochemical, differential pulse voltammetry | Secondary AB colorimetry / ELISA | Secondary AB fluorescence | Raman-active beads functionalized by specific ABs | Secondary AB fluorescence |
| **Sample Volume** | 400 µL | 20 µL | 20 µL | 700 – 2000 µL | 30 µL | 2 µL | 20 – 100 µL | 20 µL | 50 – 80 µL |
| **Detection Time** | 100 min | 120 min | 40 min | 180 min | 210 min | 90 min | 40-200 min | 60 min | 60 min |
| **Limit of Detection** | n/a | $5 \times 10^4$ / mL | n/a | $10^5$ / mL | $4.39 \times 10^3$ / mL | n/a | $10^4$ / mL | $1.6 \times 10^2$ / mL | $4{\times}10^3$ - $4{\times}10^5$ /mL depending on AB |
| **Retrieval of EV cargo** | Lysis and RNA extraction | Potentially by elution or lysis | Magnet release | Elution or lysis and RNA | Magnet release | Magnet release | Elution* | Magnet release | Potentially picking of lipid patches, lysis** |
| **Clinical Samples** | from serum | from plasma | from plasma | from plasma and serum | from serum | from plasma | from plasma | from serum | from serum |

* needs higher concentration of EVs ($10^9$ / mL) or larger sample volumes (10mL) to obtain enough material for downstream analysis
** retainment of RNA on the lipid arrays shown by fluorescence staining

[1] S.S. Kanwar, C.J. Dunlay, D.M. Simeone, and S. Nagrath, Lab Chip **14**, 1891 (2014).
[2] P. Zhang, M. He, and Y. Zeng, Lab Chip **16**, 3033 (2016).
[3] Z. Zhao, Y. Yang, Y. Zeng, and M. He, Lab Chip **16**, 489 (2016).
[4] E. Reátegui, K.E. van der Vos, C.P. Lai, M. Zeinali, N.A. Atai, B. Aldikacti, F.P. Floyd, A. H. Khankhel, V. Thapar, F.H. Hochberg, L. V. Sequist, B. V. Nahed, B. S. Carter, M. Toner, L. Balaj, D. T. Ting, X.O. Breakefield, and S.L. Stott, Nat. Commun. **9**, 175 (2018).
[5] H. Xu, C. Liao, P. Zuo, Z. Liu, and B.-C. Ye, Anal. Chem. **90**, 13451 (2018).
[6] W. Chen, H. Li, W. Su, and J. Qin, Biomicrofluidics **13**, 054113 (2019).
[7] P. Zhang, X. Zhou, M. He, Y. Shang, A.L. Tetlow, A.K. Godwin, and Y. Zeng, Nat. Biomed. Eng. **3**, 438 (2019).
[8] Y. Wang, Q. Li, H. Shi, K. Tang, L. Qiao, G. Yu, C. Ding, and S. Yu, Lab Chip **20**, 4632 (2020).
